# Physical Activity, Genetic Susceptibility, and the Risk of Latent Autoimmune Diabetes in Adults and Type 2 Diabetes

**DOI:** 10.1210/clinem/dgaa549

**Published:** 2020-08-24

**Authors:** Rebecka Hjort, Emma Ahlqvist, Tomas Andersson, Lars Alfredsson, Per-Ola Carlsson, Valdemar Grill, Leif Groop, Mats Martinell, Elin Pettersen Sørgjerd, Tiinamaija Tuomi, Bjørn Olav Åsvold, Sofia Carlsson

**Affiliations:** 1 Institute of Environmental Medicine, Karolinska Institutet, Stockholm, Sweden; 2 Department of Clinical Sciences in Malmö, Clinical Research Centre, Lund University, Malmö, Sweden; 3 Center for Occupational and Environmental Medicine, Region Stockholm, Stockholm, Sweden; 4 Department of Medical Sciences, Uppsala University, Uppsala, Sweden; 5 Department of Clinical and Molecular Medicine, NTNU, Norwegian University of Science and Technology, Trondheim, Norway; 6 Institute for Molecular Medicine Finland FIMM, Helsinki University, Helsinki, Finland; 7 Department of Public Health and Caring Sciences, Uppsala University, Uppsala, Sweden; 8 HUNT Research Centre, Department of Public Health and Nursing, NTNU, Norwegian University of Science and Technology, Trondheim, Norway; 9 Department of Endocrinology, St. Olavs Hospital, Trondheim University Hospital, Trondheim, Norway; 10 Division of Endocrinology, Abdominal Center, Helsinki University Hospital, Research Program for Diabetes and Obesity, University of Helsinki, and Folkhälsan Research Center, Helsinki, Finland; 11 K.G. Jebsen Center for Genetic Epidemiology, Department of Public Health and Nursing, NTNU, Norwegian University of Science and Technology, Trondheim, Norway

**Keywords:** physical activity, gene-environment interaction, LADA, latent autoimmune diabetes in adults, type 2 diabetes, population-based

## Abstract

**Purpose:**

Physical activity (PA) has been linked to a reduced risk of type 2 diabetes by reducing weight and improving insulin sensitivity. We investigated whether PA is associated with a lower incidence of latent autoimmune diabetes in adults (LADA) and whether the association is modified by genotypes of human leukocyte antigen (HLA), transcription factor 7-like 2 (*TCF7L2*)-rs7903146, or the fat mass and obesity-associated gene, *FTO*-rs9939609.

**Methods:**

We combined data from a Swedish case-control study and a Norwegian prospective study including 621 incident cases of LADA and 3596 cases of type 2 diabetes. We estimated adjusted pooled relative risks (RRs) and 95% CI of diabetes in relation to high (≥ 30 minutes of moderate activity 3 times/week) self-reported leisure time PA, compared to sedentariness.

**Results:**

High PA was associated with a reduced risk of LADA (RR 0.61; CI, 0.43-0.86), which was attenuated after adjustment for body mass index (BMI) (RR 0.90; CI, 0.63-1.29). The reduced risk applied only to noncarriers of HLA-*DQB1* and -*DRB1* (RR 0.49; CI, 0.33-0.72), *TCF7L2* (RR 0.62; CI, 0.45-0.87), and *FTO* (RR 0.51; CI, 0.32-0.79) risk genotypes. Adjustment for BMI attenuated but did not eliminate these associations. For type 2 diabetes, there was an inverse association with PA (RR 0.49; CI, 0.42-0.56), irrespective of genotype.

**Main Conclusions:**

Our findings indicate that high PA is associated with a reduced risk of LADA in individuals without genetic susceptibility.

Physical activity (PA) is associated with a reduced risk of type 2 diabetes, as shown both in observational (1) and intervention studies (2). The physiological pathways potentially linking PA to improved glucose uptake involve beneficial effects on body weight as well as direct effects on insulin sensitivity (3). PA has also been associated with improved insulin secretion as a result of decrease in β-cell lipid accumulation (4). Whether PA may also prevent or postpone autoimmune forms of diabetes is not clear.

Latent autoimmune diabetes in adults (LADA) is a common form of diabetes, characterized by mild autoimmunity and slower progression to insulin dependence than what is seen in type 1 diabetes, together with features of type 2 diabetes such as insulin resistance (5) and overweight (6). Similar to type 2 diabetes, PA might also prevent LADA through reducing overweight and insulin resistance or improving insulin secretion. On the other hand, PA might also act through a pathway involving autoimmunity; an in vitro study of children found that high PA may have positive effects on the immune system by altering autoantigen-induced immune activity (7). Support for a protective effect of PA on LADA risk comes from a small study based on 11 years’ follow-up of the Nord-Trøndelag Health Study (HUNT) (8), but that study included only 81 cases and could not address underlying mechanisms. We are not aware of any other study on the association between PA and the risk of autoimmune diabetes in adults or in children.

Similar to type 1 diabetes, human leukocyte antigen (HLA) genotypes, particularly in the HLA-*DRB1* and *DQB1* genes, confer a high risk of LADA (9-11). LADA has also been linked to genes associated with type 2 diabetes, for example, risk variants of the transcription factor 7-like 2 (*TCF7L2*) gene (12-14) and in one study, with the fat mass and obesity-associated (*FTO)* gene (15). We have recently shown that overweight and high-risk genotypes of HLA, *TCF7L2*, and *FTO* interact in relation to the risk of LADA (16). At the same time, the effect of *FTO* on risk of obesity is attenuated by PA (17). Hence it can be hypothesized that the impact of PA on LADA risk depends on genetic susceptibility. The aim of this study was to investigate the association between PA and incidence of LADA compared with the associations regarding type 2 diabetes, as well as whether these associations are modified by HLA, *FTO*, or *TCF7L2* genotypes and potential underlying mechanisms. We used updated data from the prospective HUNT study, including 22 years of follow-up and more than twice as many cases of LADA and type 2 diabetes as the previous study (8), and newly collected data from a Swedish case-control study with incident cases.

## Materials and Methods

### The ESTRID study

#### Study population. 

**Figure 1.**
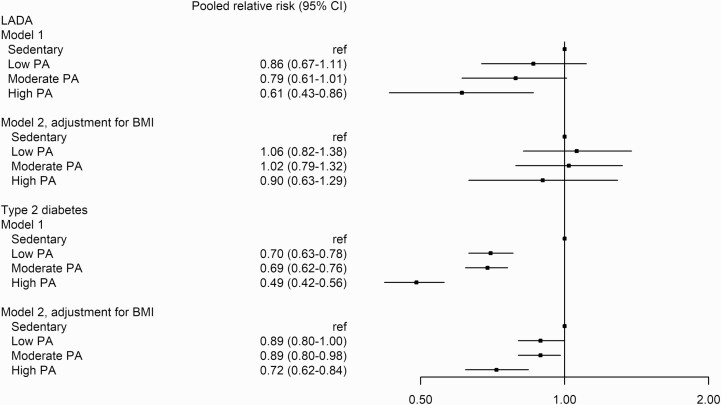
Pooled relative risks (RRs) and 95% CI of latent autoimmune diabetes in adults (LADA) and type 2 diabetes in relation to physical activity (PA) in 4 groups (sedentary vs low, moderate, or high activity). Model 1 is adjusted for age, sex, smoking, and family history of diabetes. Model 2 is adjusted for age, sex, smoking, family history of diabetes, and body mass index.

ESTRID (Epidemiological Study of Risk Factors for LADA and Type 2 Diabetes) (18) (Extra Supplemental Material [ESM] Fig. 1) (19) is an ongoing population-based case-control study from Sweden created with the purpose of studying lifestyle factors in relation to incidence of LADA and type 2 diabetes. ESTRID is nested in ANDIS (All New Diabetics in Scania), an extensive diabetes registry and biobank, aiming at classifying all new cases of diabetes within Scania County (20) and ANDiU (All New Diabetics in Uppsala County), a similar registry in the County of Uppsala. To the ESTRID study we have invited all incident cases of LADA and a random sample of type 2 diabetes cases registered in ANDIS (95% of cases) since 2010 and ANDiU (5% of cases) since 2012 and collected questionnaire information (within a median of 5 months after diagnosis for cases) in addition to clinical and genetic information obtained from the diabetes registries. Control individuals without diabetes (age ≥ 35 years) are randomly sampled through the national population register (6/LADA case) and matched to cases through incidence density matching (21). The controls provide questionnaire information but there is no genetic information. For the purpose of the genetic analyses of the present paper, we have therefore used controls from the EIRA study (Epidemiological Investigation of Rheumatoid Arthritis), which uses a similar questionnaire in addition to a biobank (22). These controls, which we hereafter will refer to as “genetic controls,” are randomly selected from the Swedish population registry, free of diabetes, and matched to our cases by age and sex.

Our analytical sample includes all cases and controls with complete information on PA and all covariates, enrolled in ESTRID between 2010 and 2017 (LADA n = 474 [393 with genetic information], type 2 diabetes n = 1594 [1286 with genetic information], controls n = 1886); together with genetic control individuals who were all controls recruited to the EIRA study between 2006 and 2014 with information on PA, all covariates, and at least one of the genotypes of interest (n = 1557). All participants gave their written informed consent and the regional ethical review board in Stockholm approved the study.

#### Diabetes classification. 

Patients were diagnosed within the health care system of Scania and classified as LADA if they were 35 years or older, glutamic acid decarboxylase antibody (GADA) positive (≥ 10.7 IU/mL, enzyme-linked immunosorbent assay, RSR Limited), and had fasting serum C-peptide levels of 0.2 nmol/L or greater (IMMULITE 2000 [Siemens Healthcare Diagnostics Product Ltd]) or 0.3 nmol/L or greater (Cobas e 601 analyzer, Roche Diagnostics). Patients with type 2 diabetes were GADA negative and had C-peptide greater than 0.6 nmol/L (IMMULITE) or greater than 0.72 nmol/L (Cobas). At the GADA cutoff point 10.7 IU/mL specificity was 98% and sensitivity 84% (23). Homeostasis model assessment (HOMA)–measure of insulin resistance (IR) and HOMA-β (estimating β-cell function) were calculated based on fasting plasma glucose and serum C-peptide (24).

#### Genetic information. 

Blood samples for genotyping of patients were analyzed at the Clinical Research Center in Malmö, Sweden, using iPlex Gold Technology (Sequenom). Missing genotypes were imputed for a subset using Infinium CoreExome v1.1 (Illumina) based on the Haplotype Reference Consortium (http://www.haplotype-reference-consortium.org/; version r1.1 2016) reference panel. Controls were genotyped based on genome-wide association study data generated through an Illumina Global Screening array or an Infinium Illumina 300K immunochip custom array. Three single-nucleotide variations (SNVs, formerly single-nucleotide polymorphisms [SNPs]) (rs3104413, rs2854275, rs9273363) were used to define HLA-*DRB1* and *DQB1* genotypes associated with high (DR4/4, DR3/3, DR3/4, DR3/4-DQ8, DR4/4-DQ8, DR4/X-DQ8) or low/intermediate (DR4/X, DR3/X, DRX/X, DR4-DQ7) risk for type 1 diabetes. The selection of HLA-genotyped SNVs was based on a validated method with an overall accuracy of 99.3% (25). The *TCF7L2* gene was genotyped with SNV rs7903146 and the *FTO* gene with SNV rs9939609.

### The HUNT study

#### Study population. 

In the northern part of the Norwegian county of Trøndelag, 3 health surveys were conducted between 1984 and 2008 (HUNT1, 1984-1986; HUNT2, 1995-1997; HUNT3, 2006-2008) with the aim of studying a range of health outcomes, including diabetes (26) (ESM Fig. 2) (19). The surveys targeted all residents aged 20 years or older, and participants completed a questionnaire with items on health and lifestyle and attended in a clinical examination that included blood sampling and measurements of height and weight. Based on these cross-sectional studies, we have formed a cohort consisting of those who participated in at least 2 surveys, and at baseline were free of diabetes and with information on PA and all covariates (n = 54 258 [46 567 with genetic information]). Informed consent was given by all participants and the study was approved by the Norwegian Data Protection Authority and the Regional Committee for Medical and Health Research Ethics.

#### Diabetes classification. 

Incident cases of diabetes were identified by self-reporting at HUNT2 or HUNT3. A previous validation study shows that these self-reports correctly classify more than 95% of patients (27). Fasting blood samples were collected at follow-up in HUNT (median, 4 years after diagnosis) and based on this, all patients with diagnosis at age 35 years or older were classified as having LADA (n = 147) if they were GADA positive (≥ 43 IU/mL, immunoprecipitation radioligand assay Novo Nordisk) and as having type 2 diabetes if they were GADA negative (n = 2002). At the positive cutoff for GADA, specificity was 1.00 and sensitivity 0.64 according to the IASP (Islet Autoantibody Standardization Program) 2003 workshop. This LADA definition implies that some patients with adult-onset type 1 diabetes will be included. As discussed previously, the proportion of such patients is likely to be small (6). HOMA indices were calculated based on fasting serum C-peptide and fasting serum or whole-blood glucose, measured as described previously.

#### Genetic information. 

DNA samples were analyzed at the NTNU Genomic Core Facility in Trondheim using HumanCoreExome arrays (Illumina, https://www.ntnu.no/hunt/gwas-data) and genotyped for SNVs associated with HLA-*DRB1* and HLA-*DQB1* (rs2854275, rs9273363, rs9272346), *TCF7L2* (rs7903146), and *FTO* (rs9939609). Imputation was computed with Minimac3 (v2.0.1, http://genome.sph.umich.edu/wiki/Minimac3) from a customized Haplotype Reference consortium release 1.1 (HRC v1.1). For the HLA analysis, 3 SNVs (rs2854275, rs9273363, rs9272346) previously associated with LADA and type 1 diabetes (20, 25, 28, 29) were chosen based on the availability in our study sample. Out of these, rs2854275 and rs9273363 were also used in ESTRID. Participants were classified as having an HLA high-risk genotype if carrying at least one of the risk variants inferring DR3-DQ2 haplotype; rs2854275 (TT/TG) and rs9272346 (AA) or DR4-DQ8 haplotype; rs9273363 (AA), and the others were considered to have low/moderate risk.

#### Physical activity. 

In ESTRID, information on PA (see Appendix for detailed information on all questions in ESTRID and HUNT) (19) was obtained by a question on average leisure time PA during the previous year, that is, the year prior to diagnosis in diabetic patients. There were 4 response alternatives, ranging from 1) sedentary; moderate PA for less than 2 hours per week, 2) low PA; moderate PA, mostly without sweating for 2 or more hours per week, 3) moderate PA; PA that makes you sweat 1 or 2 times per week, each time for 30 minutes or more, and 4) high PA; PA that makes you sweat 3 or more times per week, each time for 30 minutes or more. Answers to this question have been shown to correlate with accelerometer data recorded during a 7-day period (r = 0.37; *P < *.001) and to work well in correctly discriminating between activity groups (*P* for trend < .001) (30). The questions on PA in the HUNT study were more detailed and had to be harmonized to fit with the 4 response alternatives in the ESTRID questionnaire. In HUNT1 leisure time PA was measured by the question “How often do you exercise?” with 5 response options ranging from never to nearly every day (31). To fit with the ESTRID data, categories 1 to 2 were collapsed and PA was classified as 1) sedentary; exercise less than 1 time per week; 2) low; 1 time per week, 3) moderate; 2 or 3 times per week and 4) high; nearly every day. In HUNT2, there were separate questions on average number of hours of weekly light and hard activity during the last year with 4 response options ranging from 0 to 3 or more hours per week (32). The answers to these 2 questions were combined to indicate average PA in 4 categories; 1) sedentary; 2 hours or less of light activity a week and no hard activity, 2) low; 3 hours or more of light activity a week and no hard activity, 3) moderate; 1 to 2 hours of hard activity a week, and 4) high; 3 hours or more of hard activity a week. In addition, we used data from HUNT1 to obtain a more detailed assessment of PA based on information frequency, duration (< 15, 15-30, 31-60, or ≥ 60 minutes) and intensity (light, moderate, or high) of the activity. This information was combined into a summary score by a previously described method (33).

#### Covariates. 

ESTRID: Body mass index (BMI) was calculated based on self-reported weight and height as kilograms divided by meters squared (kg/m^2^). For the patients, this information has been validated against their medical records with a high degree of accuracy (r = 0.92, *P *< .0001) (6). There were questions on lifetime tobacco use, alcohol consumption, and highest attained education. Family history of diabetes (FHD) based on questions on diabetes in first-degree relatives was available only in the ESTRID study and not for the genetic controls from the EIRA study. HUNT: BMI was based on measures of height and weight from the baseline investigation. The baseline questionnaires provided information on highest attained education, FHD, smoking history, and alcohol consumption.

#### Statistical analyses. 

Descriptive statistics were compared with χ ^2^ (proportions), Kruskal-Wallis (medians) and *t* (means) tests and 2-sided *P* values were calculated. The association between PA or genotype with LADA or type 2 diabetes was assessed by odds ratios (ORs) with 95% CI estimated by conditional logistic regression for the ESTRID case-control data. ESTRID controls were used in the overall analyses of PA (to gain power and to be able to adjust for FHD), and the “genetic controls” served as reference in all analyses including genetic data (because we did not have genetic information for the ESTRID controls). Corresponding hazard ratios (HRs) with CIs were calculated by proportional Cox regression for the HUNT prospective data, modeled with age as the underlying time scale. Participants in HUNT were followed from age at baseline until age at onset of diabetes, death, or age at follow-up (in HUNT2 or HUNT3). For convenience, we use the term relative risk (RR) to describe the effect estimates. In addition to presenting study-specific RRs, we performed a meta-analysis by pooling the risk-estimates in ESTRID and HUNT with the inverse variance method (34). These pooled RRs will be presented throughout the paper unless otherwise stated. In the main analysis, PA was divided into 4 categories (sedentary vs low, moderate, or high activity) and into 2 groups (sedentary vs active [low, moderate or high activity]) when stratified by genotypes of HLA (high vs low/intermediate risk), *TCF7L2*-rs7903146 (TT/TC vs CC), and *FTO*-rs9939609 (AA/AT vs TT). Model 1 was adjusted for age, sex (the matching variables in the genetic analyses), and smoking (never, former, current) and, except in the analyses including genetic information, by FHD (yes vs no). Model 2 was additionally adjusted for BMI, which was considered a potential mediator. Results from model 1 are presented in “Results” unless otherwise specified. Additional adjustment for alcohol consumption (abstainers, low, moderate, or high consumers) and education (primary school, upper secondary school, or university) did not change the effect estimates; these variables were therefore not included in the final models. We conducted sensitivity analyses in HUNT based on a stricter definition of LADA (ie, no insulin treatment during the first year of diagnosis) and a PA summary score including intensity and duration of the PA, available for a subset of the population. In ESTRID we repeated the main analysis of PA and LADA/type 2 diabetes using genetic controls to assess whether results were comparable to those based on controls from ESTRID. Statistical Analysis Software (SAS) 9.4 (SAS Institute) was used for all statistical computations.

## Results

### Characteristics

In ESTRID, individuals with LADA were younger and leaner, more likely to be treated with insulin, had lower insulin secretion, and were less insulin resistant than those with type 2 diabetes. Comparing controls from ESTRID to the genetic controls from EIRA showed that the latter were more likely to be female (because rheumatoid arthritis is more common among women); this was handled by matching to the diabetes cases. In HUNT, LADA patients were less likely to have first-degree FHD but were more often treated with insulin and had lower levels of C-peptide than patients with type 2 diabetes (Table 1).

**Table 1.** Characteristics of the participants

Characteristics	HUNT				ESTRID				
	No diabetes	T2D	LADA	*P* ^*a*^	Controls	Genetic controls	T2D	LADA	*P* ^*a*^
No. of individuals	54 258	2002	147		1886	1557	1594	474	
Men, %	46.7	52.7	48.3	.30	47.8	26.5	61.2	53.6	.003
Age at diagnosis/index, mean (SD), y	–	60.9 (10.9)	59.9 (11.1)	.30	58.4 (13.5)	57.6 (9.8)	63.2 (10.3)	59.0 (12.3)	< .001
Age at baseline (HUNT), mean (SD), y	48.3 (15.8)	54.8 (11.0)	54.4 (11.2)	.69	–	–	–	–	–
BMI, mean (SD), kg/m^2^	25.5 (3.8)	29.8 (4.5)	29.2 (4.9)	.17	25.9 (4.2)	25.4 (4.1)	31.2 (5.4)	28.1 (5.3)	< .001
Any first-degree FHD, %	24.2	57.1	48.3	.0379	24.3	–	49.2	43.0	.02
FHD-T2D, %	–	–	–	–	22.6	–	47.2	35.7	< .001
FHD-T1D, %	–	–	–	–	2.4	–	4.6	10.1	< .001
With insulin treatment, %	–	15.2	43.5	< .001	22.65	–	5.9	43.3	< .001
GADA, median (IQR), IU/mL	–	–	134 (59-581)	–	2.58	–	-	226 (28-250)	–
C-peptide, median (IQR) nmol/L	-	0.86 (0.60-1.19)	0.57 (0.20-0.98)	< .001	–	–	1.20 (0.96-1.60)	0.69 (0.43-1.20)	< .001
HOMA-β, median (IQR)	–	64.5 (43.0-92.2)	59.0 (36.6-87.4)	.41	–	–	68.8 (43.5-93.7)	37.9 (14.6-67.9)	< .001
HOMA-IR, median (IQR)	–	2.20 (1.60-3.20)	2.10 (1.20-2.90)	.11	–	–	3.60 (2.70-4.80)	2.80 (1.80-4.40)	< .001
HLA high-risk^*b*^, %	42.5	40.7	60.3	< .001	–	33.6	31.3	61.1	< .001
TT/TC in *TCF7L2*-rs7903146, %	44.2	54.9	44.3	.02	–	46.3	52.1	51.9	.93
AA/AT in *FTO*-rs9939609, %	65.9	69.2	76.3	.08	–	64.3	67.4	66.2	.68

Clinical information was available for 98% of patients in ESTRID (LADA n = 463, T2D n = 1558) and 70% of patients in HUNT (LADA n = 118, T2D = 1401). Genetic information was available for 79% of the patients in ESTRID (LADA n = 393, T2D n = 1241) and 95% of the patients in HUNT (LADA n = 131, T2D n = 1901).

Abbreviations: BMI, body mass index; ESTRID, Epidemiological Study of Risk Factors for LADA and Type 2 Diabetes; FHD, family history of diabetes; *FTO*, fat mass and obesity-associated gene; GADA, glutamic acid decarboxylase antibody; HLA, human leukocyte antigen; HOMA-β, homeostasis model assessment–estimating β-cell function; HOMA-IR, homeostasis model assessment–insulin resistance; HUNT, Nord-Trøndelag Health Study; IQR, interquartile range; LADA, latent autoimmune diabetes in adults; T1D, type 1 diabetes; T2D, type 2 diabetes; *TCF7L2*, transcription factor 7-like 2 gene.

^*a*^P for difference between LADA and T2D.

^*b*^HLA high-risk genotypes: DR4/4, DR3/3, DR3/4, DR3/4 to DQ8, DR4/4 to DQ8, or DR4/X to DQ8 in ESTRID and DR3 to DQ2 or DR4 to DQ8 in HUNT.

### Genotype and risk of latent autoimmune diabetes in adults and type 2 diabetes

As previously reported in these populations (16), LADA was strongly associated with HLA high-risk genotypes RR_pooled_ 2.59 (95% CI, 2.08-3.22) and tended to be associated with *TCF7L2*_rs7903146_ (RR_pooled_ 1.18, 95% CI, 0.96-1.43) and *FTO*_rs9939609_ (RR_pooled_ 1.24, 95% CI, 1.00-1.54). Type 2 diabetes was associated with *TCF7L2* (RR_pooled_ 1.46, 95% CI, 1.35-1.59) and *FTO (*RR_pooled_ 1.15, 95% CI 1.06-1.26) but not with HLA (RR_pooled_ 0.94, 95% CI, 0.86-1.02). Adjusting for the other genotypes did not change these estimates (data not shown).

### Physical activity, latent autoimmune diabetes in adults, and type 2 diabetes

High PA, corresponding to exercising for at least 30 minutes on at least 3 occasions per week, was associated with reduced incidence of LADA (RR_pooled_ 0.61, 95% CI, 0.43-0.86), but the association was eliminated after further adjustment for BMI (RR_pooled_ 0.90, 95% CI, 0.63-1.29) (see Fig. 1, ESM Table 1) (19). Incidence of type 2 diabetes was also reduced in individuals with high PA (RR_pooled_ 0.49, 95% CI, 0.42-0.56), and this association was attenuated but remained after adjustment for BMI (RR_pooled_ 0.72, 95% CI, 0.62-0.84) (see Fig. 1). No appreciable differences were seen in the study-specific analyses even though numbers were smaller and CIs wider, that is, the most active individuals had the lowest incidence of diabetes (ESM Table 1) (19). Results were similar when PA was analyzed in 2 groups (active vs sedentary) (ESM Table 2) (19).

### Stratification by genotype

**Figure 2.**
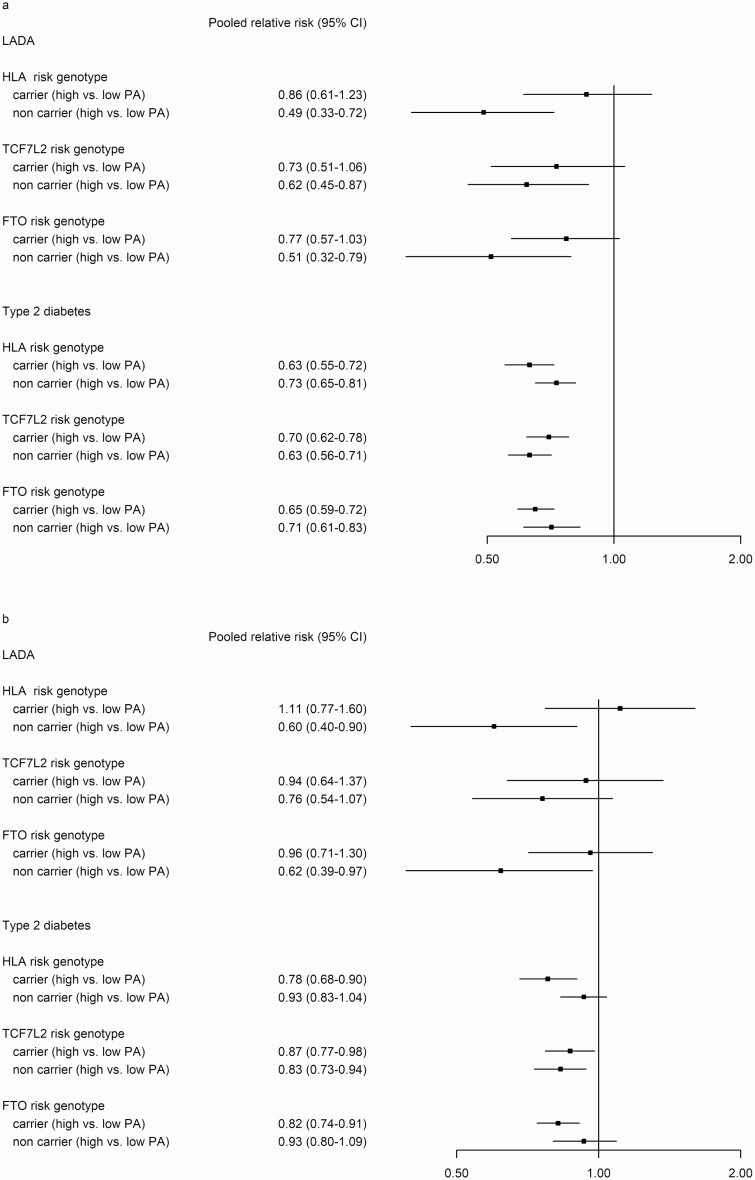
Pooled relative risks (RRs) and 95% CI of latent autoimmune diabetes in adults (LADA) and type 2 diabetes in relation to physical activity (PA) in 2 groups (active vs sedentary) stratified by high and low/intermediate risk genotypes of human leukocyte antigen (HLA), transcription factor 7-like 2 (*TCF7L2*), and fat mass and obesity (*FTO*)-associated genes. Adjusted for A, age, sex, smoking; and additionally for B, body mass index.

Stratification by genotypes indicated that the reduced incidence of LADA associated with PA (active vs inactive) applied only to individuals with low-risk genotypes of HLA; RR_pooled_ 0.49 (95% CI, 0.33-0.72), *FTO*; RR_pooled_ 0.51 (95% CI, 0.32-0.79), and *TCF7L2*; RR_pooled_ 0.62 (95% CI, 0.45-0.87), whereas no association was seen in carriers of the high-risk variants (see Fig. 2). Furthermore, the reduced incidence associated with PA remained even after adjustment for BMI in low-risk carriers of HLA and *FTO* genotypes. For type 2 diabetes, the association with PA did not appear to be modified by either *TCF7L2*, *FTO*, or HLA (see Fig. 2). Study-specific estimates were similar, that is, high PA was associated with a reduced incidence of LADA primarily in individuals without the high-risk genetic variants both in ESTRID and HUNT (ESM Table 3) (19). The results were also comparable when we analyzed the incidence of LADA/type 2 diabetes in relation to PA in four categories, stratified by genotype (ESM Table 4) (19).

### Clinical characteristics of latent autoimmune diabetes in adults and type 2 diabetes by level of physical activity

In ESTRID, sedentary compared to active LADA patients had a more type-2 like phenotype with higher mean BMI, median HOMA-IR, and HOMA-β (see Table 2). In HUNT, numbers were small, and no clear differences were seen between sedentary and active LADA patients (data not shown). For type 2 diabetes patients in ESTRID, sedentary vs active individuals had higher levels of BMI, C-peptide, and HOMA-IR.

**Table 2.** Characteristics of individuals with latent autoimmune diabetes in adults and type 2 diabetes by level of physical activity

	LADA			T2D		
	Sedentary	Active	*P*	Sedentary	Active	*P*
No. of individuals	82	392		380	1214	
Men, %	65.9	51.0	.01	62.1	61.0	.69
Age at diagnosis, mean (SD), y	57.2 (11.1)	59.5 (12.4)	.12	61.2 (10.5)	63.7 (10.1)	< .001
BMI, mean (SD), kg/m^2^	31.2 (6.4)	27.6 (5.1)	< .001	32.9 (6.1)	30.6 (5.0)	< .001
GADA, median (IQR), IU/mL	113 (21-250)	238 (32-250)	.12	–	–	–
C-peptide, median (IQR), nmol/L	0.98 (0.59-1.44)	0.65 (0.40-1.04)	< .001	1.40 (1.00-1.70)	1.20 (0.95-1.50)	< .001
HOMA-β, median (IQR)	51.1 (19.3-85.0)	36.6 (13.1-65.6)	.01	69.8 (43.7-97.9)	68.7 (42.8-91.9)	.34
HOMA-IR, median (IQR)	3.60 (2.40-5.50)	2.60 (1.80-4.20)	.003	4.00 (2.90-4.90)	3.50 (2.70-4.80)	.006

Data from the ESTRID (Epidemiological Study of Risk Factors for LADA and Type 2 Diabetes) study. Sedentary individuals are those with less than 2 hours of moderate activity per week. Active individuals are those with 2 or more hours of moderate activity per week.

Abbreviations: BMI, body mass index; GADA, glutamic acid decarboxylase antibody; HOMA-β, homeostasis model assessment–estimating β-cell function; HOMA-IR, homeostasis model assessment–insulin resistance; IQR, interquartile range; LADA, latent autoimmune diabetes in adults; T2D, type 2 diabetes.

### Sensitivity analyses

Separate analyses of the overall association between PA and LADA/type 2 diabetes using genetic controls as reference (ESM Table 5) (19) indicated similar results as those based on controls sampled within the ESTRID study (ESM Table 1) (19). When we used a more detailed assessment of PA, including duration and intensity of the activity, that was available for a subset of the HUNT population, the association between PA and LADA/type 2 diabetes was similar to that seen in the overall analyses (ESM Table 6) (19). Separate analysis of the HUNT data with a stricter LADA definition, that is, no insulin treatment, also indicated similar results regarding the association with PA as the main analyses (ESM Table 7) (19).

## Discussion

High PA corresponding to at least 30 minutes of moderate activity, 3 times per week, was associated with a 40% lower incidence of LADA. The lower incidence was eliminated after adjustment for BMI, suggesting that a potential effect is mediated by reduced adiposity, alternatively that BMI is confounding the association between LADA and PA. Separate analyses in individuals with genetic susceptibility to HLA, *TCF7L2*, and *FTO* genotypes reveal that high PA is associated with lower incidence of LADA only in individuals who do not carry high-risk variants, and this association was seen even after adjustment for BMI. The association between PA and type 2 diabetes was stronger than between PA and LADA, and incidence was lower in those with high PA irrespective of the genetic risk variants.

Our findings indicate that PA may reduce the incidence of LADA through the same mechanism linking PA to type 2 diabetes, namely through beneficial effects on body weight and improved insulin sensitivity (1, 3). In contrast, our data did not support that PA inhibits autoimmunity because active individuals had higher GADA levels, worse β-cell function, and lower levels of C-peptide than those who were sedentary. We can only speculate on why PA is associated with a lower incidence of LADA only in individuals without genetic susceptibility to diabetes; the HLA-*DRB1* and HLA-*DQB1* genotypes are the main risk factors for β-cell autoimmunity and loss of β-cell function leading to type 1 diabetes (35). Risk variants of *TCF7L2* have also been linked to reduced insulin secretion (36). One interpretation of our findings is therefore that any beneficial effects on body weight and insulin sensitivity conferred by PA will have minor influence on the risk of developing diabetes in individuals carrying these risk variants, because lack of insulin may be the main driver in disease development. Why the same results apply to carriers of the *FTO* risk variant is not clear. Importantly, these results should be interpreted with caution; this is the first study to address the association between PA and LADA in relation to genetic susceptibility, and extensions and replications are clearly warranted. It should also be noted that the analyses of PA by genotype were hampered by relatively low numbers and overlapping CIs. Still, these findings fit with previous observations that high consumption of sweetened beverages only increases the risk of LADA in individuals without HLA risk genotypes (37). This would suggest that the influence of environmental factors in the etiology of LADA, and thus opportunities for lifestyle interventions, are larger in individuals without genetic susceptibility to diabetes. We have recently observed that the combination of HLA risk genotypes and overweight confers an 8-fold increased risk of LADA (16). It seems reasonable that a positive effect of PA on body weight and insulin sensitivity may not be enough to counteract the influence of such strong risk factors.

Consistent with previous studies (1), we find a 50% lower incidence of type 2 diabetes in individuals engaging in high PA vs those who are sedentary, and the risk reduction seemed equally strong in carriers and noncarriers of the *TCF7L2* and *FTO* risk variants. Previous data on the topic are limited but this observation fits with results from the EPIC InterAct study; no synergistic effect was found between PA and a genetic risk score including both *TCF7L2* and *FTO* (38). Previous studies also show that PA attenuates the excess risk of obesity conferred by risk variants of *FTO* (17). These findings indicate that lifestyle interventions can reduce the incidence of type 2 diabetes in genetically predisposed individuals, which is in line with previous findings from the Diabetes Prevention Program (39). The different results seen for LADA and type 2 diabetes in terms of effect modification by genetic susceptibility may be attributed to the fact that insulin resistance plays a relatively smaller role in the pathogenesis of LADA.

This study has several strengths, including the large number of LADA patients (> 600), the detailed information on lifestyle, clinical, and genetic factors, validated questions on PA (30-32), and we also had the opportunity to replicate the analyses in 2 populations: the Norwegian prospective HUNT study and ESTRID, which is a Swedish case-control study that recruits incident cases through from the ANDIS biobank. A limitation was that the PA questions differed in their phrasing and number of response options across studies, which hampers comparability. A further limitation is that the PA questions in ESTRID could not distinguish between PA of different intensity; still, sensitivity analyses in HUNT, including intensity and duration of the activity, showed similar results. In the ESTRID study, the PA information was reported retrospectively and may therefore be afflicted with recall bias if cases report differently from controls. If individuals with diabetes tend to underestimate their previous PA, this will lead to an overestimation of an inverse association between PA and LADA/type 2 diabetes. In this context it is important to note that results based on ESTRID were similar to those based on data from the prospective HUNT study, in which information on PA was obtained several years prior to diagnosis. With prospective data any misclassification of PA can be expected to be independent of future disease status and therefore lead to diluted rather than overestimated associations. A long time between the PA assessment and diabetes onset will also lead to misclassification of PA and diluted associations. Importantly, our findings regarding PA and type 2 diabetes based on ESTRID as well as HUNT were in line with a large number of previous prospective studies (1, 2) supporting the validity of our data. We had the opportunity to adjust for a range of potential confounders, but unmeasured confounding or crude assessment of confounders, including self-reported information on BMI in ESTRID, could still have influenced the results.

The definition of LADA is in line with commonly used criteria (5) but the criterion for distinguishing LADA from type 1 diabetes patients differed across studies; in ESTRID we used C-peptide levels as an indicator of remaining insulin production, whereas we used lack of insulin treatment within the first year after diagnosis in HUNT (sensitivity analysis). The specificity of the GADA assay was high but some type 2 diabetes patients may have been misclassified as having LADA, and this could contribute to an association with PA. In HUNT, GADA was measured on participation in the survey rather than at time of diagnosis, which could have been several years earlier. Because GADA can fade over time (40), some false-negative LADA patients may have been included in the type 2 diabetes group. However, GADA has proved to be more stable in LADA than in type 1 diabetes patients (5). Moreover, none of those who developed type 2 diabetes between HUNT2 and HUNT3 were GADA positive at HUNT2 (41). Notably, there was a strong association between the HLA high-risk genotypes and LADA, but not with type 2 diabetes, indicating that the rate of misclassified patients is low. GADA was the only available autoantibody, but it has been shown to be by far the most prevalent autoantibody in LADA (40, 42). Using controls borrowed from another study is an additional drawback. The validity of this approach is supported by the finding of similar associations between PA and LADA/type 2 diabetes irrespective of whether the original controls or the genetic controls served as reference. Finally, it is known from previous studies (43) that LADA populations are heterogeneous in terms of clinical characteristics, ethnicity, and genetic makeup, and it remains to be seen whether these findings can be generalized to populations outside Scandinavia.

In conclusion, this study indicates that PA may prevent or postpone LADA through similar mechanistic pathways linking PA to type 2 diabetes but only in individuals with low genetic risk. The risk reduction does not seem to apply to individuals with genetic susceptibility in whom a potential beneficial effect of PA may not be enough to prevent LADA from developing.

## Data Availability

Restrictions apply to the availability of data generated or analyzed during this study to preserve patient confidentiality or because they were used under license. The corresponding author will on request detail the restrictions and any conditions under which access to some data may be provided.

## Abbreviations

ANDIS: All New Diabetics in Scania
ANDiU: All New Diabetics in Uppsala
BMI: body mass index
EIRA: Epidemiological Investigation of Rheumatoid Arthritis
ESTRID: Epidemiological Study of Risk Factors for LADA and Type 2 Diabetes
FHD: family history of diabetes
FTO: fat mass and obesity-associated gene
GADA: glutamic acid decarboxylase antibody
HLA: human leukocyte antigen
HOMA-β: homeostasis model assessment–estimating β-cell function
HOMA-IR: homeostasis model assessment–insulin resistance
HUNT: Nord-Trøndelag Health Study
LADA: latent autoimmune diabetes in adults
PA: physical activity
RR: relative risk
SNV: single-nucleotide variation
TCF7L2: transcription factor 7-like 2 gene

## Additional Information

***Disclosure Summary:*** The authors have nothing to disclose.
